# Time trends in socio-economic and geographic-based inequalities in childhood wasting in Guinea over 2 decades: a cross-sectional study

**DOI:** 10.1093/inthealth/ihac002

**Published:** 2022-02-01

**Authors:** Betregiorgis Zegeye, Nicholas Kofi Adjei, Comfort Z Olorunsaiye, Bright Opoku Ahinkorah, Edward Kwabena Ameyaw, Eugene Budu, Abdul-Aziz Seidu, Sanni Yaya

**Affiliations:** HaSET Maternal and Child Health Research Program, Shewarobit Field Office, Shewarobit, Ethiopia; Department of Public Health and Policy, University of Liverpool, Liverpool, UK; Department of Public Health, Arcadia University, Glenside, PA, USA; School of Public Health, Faculty of Health, University of Technology Sydney, Ultimo, NSW, Australia; School of Public Health, Faculty of Health, University of Technology Sydney, Ultimo, NSW, Australia; Department of Population and Health, University of Cape Coast, Cape Coast, Ghana; Department of Population and Health, University of Cape Coast, Cape Coast, Ghana; School of International Development and Global Studies, Faculty of Social Sciences, University of Ottawa, 120 University Private, Ottawa, ON, K1N 6N5, Canada

**Keywords:** wasting, inequality, Guinea, DHS, MICS, global health

## Abstract

**Background:**

Today, an estimated 7.3% (50 million) of all children <5 y of age suffer from wasting, with more burden in African countries including Guinea. Investigating inequalities in childhood wasting is essential for designing efficient programs and interventions, but no related evidence exists in Guinea. This study aimed to examine the trends in the prevalence of childhood wasting and the extent of sex, socio-economic and geographic-based disparities in Guinea.

**Methods:**

Data from the 1999, 2005 and 2012 Guinea Demographic and Health Surveys and the 2016 Guinea Multiple Indicator Cluster Survey, with a total of 16 137 children <5 y of age were included for analysis. For inequality analysis, we used the 2019 updated World Health Organization Health Equity Assessment Toolkit (HEAT) software. Inequality was measured using four summary measures (difference [D], population attributable risk [PAR], ratio [R] and population attributable fraction [PAF]) for five equity stratifiers (economic status, education, place of residence, sex and subnational region). We computed 95% confidence intervals (CIs) around the points estimates to measure statistical significance.

**Results:**

The findings revealed a pro-rich (R=1.68 [95% CI 1.11 to 2.24]), pro-urban (PAR=−1.04 [95% CI −1.90 to −0.18]) and subnational region (D=8.11 [95% CI 4.85 to 11.36]) inequalities in childhood wasting across all surveys. Except in 2005, education-based disparities (PAF=−18.2 [95% CI −36.10 to −0.26]) were observed across all survey years, but not sex-based disparities. An approximately constant inequality pattern was seen across all dimensions.

**Conclusions:**

This study showed inequalities in childhood wasting in Guinea with a disproportionately higher risk of wasting among children from disadvantaged subpopulations/mothers, including uneducated, poorest/poor, rural residents and regions. Policies that target disadvantaged populations need to be considered in order to ensure social protection, access to a wholesome diet and universal and quality health services.

## Background

Childhood undernutrition, including wasting, is an important contributing factor to childhood illness and mortality in low-and middle-income countries.^1,2^ Globally, approximately half of all mortality in children <5 y of age has been linked to undernutrition.^3^

In 2019, globally, 47 million children <5 y of age were wasted, of which 14.3 million were severely wasted.^4^ Sub-Saharan Africa (SSA) accounts for one-quarter of the global childhood wasting burden,^4^ which is a major public health concern in the region.^5,6^ Guinea has not made much progress towards wasting, with 9.2% of children <5 y of age affected, which is higher than the average for the African region (6.0%)^7^ and most sub-Saharan African countries, including Rwanda (0.9%), South Africa (0.5%), Zimbabwe (3.0%), Uganda (4.1%), Mozambique (4.2%), Kenya (4.5%), Zambia (4.8%), Togo (6.2%), Nigeria (7.6%), Ghana (7.8%) and Ethiopia (8.8%).^4^

Undernutrition increases children's risk of dying from common infections^4^ and it also increases the frequency and severity of these infections.^4^ There is some evidence that undernutrition may have a long-term adverse effect in later life,^8–11^ including cognitive impairment as well as poor intellectual and physical development.^12,13^ Several factors, including community, household, environmental and cultural issues, are associated with children's undernutrition in low-and middle-income countries.^14^ Child characteristics such as sex, age and birthweight, as well as parental characteristics including education status, mothers’ body mass index, household economic status, residence and region, are significant factors associated with childhood undernutrition.^15,16^

Many efforts have been made in the nation to reduce childhood wasting, such as joining nutrition initiatives. For instance, in 28 May 2013, the Republic of Guinea joined the Scaling Up Nutrition movement with a letter of commitment from three ministers: Health, Agriculture and Social Welfare.^17^ In January 2016, the civil society alliance for nutrition in Conakry, Conseil National des Organisations de la Société Guinéènne, launched an awareness-raising initiative for nutrition.^18^ The initiative recognizes that women have an important role in sharing key nutrition messages that complement nutrition messages amplified through various media channels.^18^

However, the country has not made progress. Therefore, beyond these initiatives, it needs further investigation of other factors, including inequalities. Prior research showed that childhood wasting varies significantly by socio-economic status (SES), including household wealth status^14–17^ and maternal education.^8,13,19–22^ Socio-economic disparities in childhood wasting are prevalent in SSA,^8,13,19–22^ but little is known about gender and geographic disparities in childhood wasting. In addition, there is a dearth of evidence about inequalities related to childhood wasting in Guinea. Therefore, measuring inequalities between subgroups may inform policies, programs and practices to promote better health among the disadvantaged.^23,24^

Thus the aim of this study was to examine the magnitude and trends in the prevalence of childhood wasting among subpopulations by economic status, education, place of residence, sex and subnational regions in Guinea from 1999 to 2016. Furthermore, we assessed the dynamics of socio-economic, sex and geographic inequalities in childhood wasting in Guinea from 1999 to 2016.

## Methods

### Data source

The data for this study were from three waves of the Guinea Demographic and Health Survey (GDHS; 1999, 2005 and 2012) and one wave of the Guinea Multiple Indicator Cluster Survey (GMICS; 2016). The GDHS and GMICS were conducted by the National Institute of Statistics of the Ministry of Planning in collaboration with the United States Agency for International Development (USAID) and the United Nations Children's Fund, respectively, with technical assistance from Inner-City Fund International. GDHS and GMICS are highly comparable nationally representative data sources that permit direct comparison between them^25–27^ and samples of men and women in their reproductive age, and they provide an adequate representation of urban and rural settings. The surveys also covered all eight administrative regions (Boké, Conakry, Faranah, Kankan, Kindia, Labé, Mamou and Nzérékoré).

Both the GDHS and GMICS employed a two-stage stratified cluster sampling technique. First, clusters or enumeration areas (EAs) were selected across the entire nation from a list of EAs established in the most recent census. The second stage involved household sampling, where 25–30 households were selected in each cluster.^28–30^ The analysis was carried out on 16 137 children <5 y of age preceding the respective surveys.

### Variables and measurements

Wasting was the outcome variable and was measured as the weight-for-height z-score (WHZ) <−2 standard deviations (SDs) from the median of the World Health Organization (WHO) child growth standard.^3,4^ For children <5 y of age, a WHZ <−2 SDs from the WHO reference population was coded 1 and a WHZ between −2 SDs and +5 SDs was coded as 0.^3,4^ Children with WHZ <−5 SDs or >+5 SDs were considered as having invalid data and were excluded from the analysis. Children who were not weighed and measured and children whose values for weight and height were not recorded were excluded. Children whose month or year of birth was missing or unknown were flagged and excluded. Children whose day of birth was missing or unknown were assigned day 15. Children who were flagged for out-of-range z-scores or invalid z-scores were excluded.^31,32^

Inequality in wasting was measured using five equity stratifiers: economic status, education, place of residence, sex and subnational region. Economic status was approximated by a wealth index.^33^ The selection of these five dimensions of inequality (equity stratifiers) was because these equity stratifiers represent common sources of discrimination and can be widely applied to populations in low- and middle-income countries.^34^ In the GDHS and GMICS, the wealth index is usually computed using durable goods, household characteristics and basic services, following the methodology explained elsewhere.^34,35^

The constructed wealth index was further categorized into five quintiles: from poorest (quintile 1) to richest (quintile 5). Maternal education status was classified as no education, primary education and secondary education or more. Place of residence was classified as urban or rural and child sex was categorized as male or female. The subnational region included the eight regions in the country (Boké, Conakry, Faranah, Kankan, Kindia, Labé, Mamou and Nzérékoré).

### Statistical analyses

The analysis was conducted with the latest version of the WHO Health Equity Assessment Toolkit (HEAT) software. This software is used to investigate health inequalities within and between countries for >30 reproductive, maternal, newborn and child health indicators.^36,37^ A detailed discussion of the software is available elsewhere.^36,37^ We used this equity assessment toolkit to examine socio-economic and geographic inequalities in childhood wasting following two steps. First, the prevalence of wasting was disaggregated by five equity stratifiers (economic status, education status, place of residence, sex and subnational region) across different subpopulations. Second, inequality was assessed using four measures of inequality: difference (D), population attributable risk (PAR), population attributable fraction (PAF) and ratio (R).

R and PAF are relative measures, while D and PAR are absolute summary measures. The selection of these simple and complex summary measures was based on evidence that supports the scientific significance of using both absolute and relative measures in studies involving a single health inequality.^38^ This is deemed essential because of the likelihood of obtaining different and even contrasting conclusions,^38^ which can lead to bias-informed decisions.^38^ Details about summary measures and the methods for calculating the summary measures and subsequent interpretation adopted in this study have been described elsewhere^38,39^ and are available in Supplementary file 1. Regarding the interpretation of summary measures, if there is no inequality, D takes the value zero. Greater absolute values of D indicate higher levels of wasting inequality. Positive values of R indicate a higher concentration of wasting among the disadvantaged and negative values indicate a higher concentration among the advantaged. If there is no inequality, R takes the value one. It takes only positive values (>1 or <1). The further the value of R from 1, the higher the level of inequality. PAR and PAF take negative values for adverse health outcome indicators such as wasting. The larger the absolute value of PAR, the higher the level of inequality. PAR is zero if no further improvement can be achieved, i.e. if all subgroups have reached the same level of wasting prevalence as the reference subgroup. The trend of inequality for each summary measure was assessed by referring to the 95% confidence intervals (CIs) for the different survey years. Inequalities exist if the UIs do not overlap.^40^

### Ethical considerations

Ethical approval was not required because the study used publicly available GDHS and GMICS data. All DHS and MICS surveys are approved by Inner City Fund (ICF) International as well as an institutional review board (IRB) in the respective country to ensure that the protocols are in compliance with the US Department of Health and Human Services regulations for the protection of human subjects.

## Results

In this study, we examined the magnitude and trends in inequality in childhood wasting in Guinea from 1999 to 2016. Overall, a total of 16 137 children <5 y of age were included in all four surveys. Of these, 11 387 (70.6%) were rural residents. More than three-fourths (76.3%) of the respondents had no formal education and 7865 (48.7%) were female.

**Figure 1.**
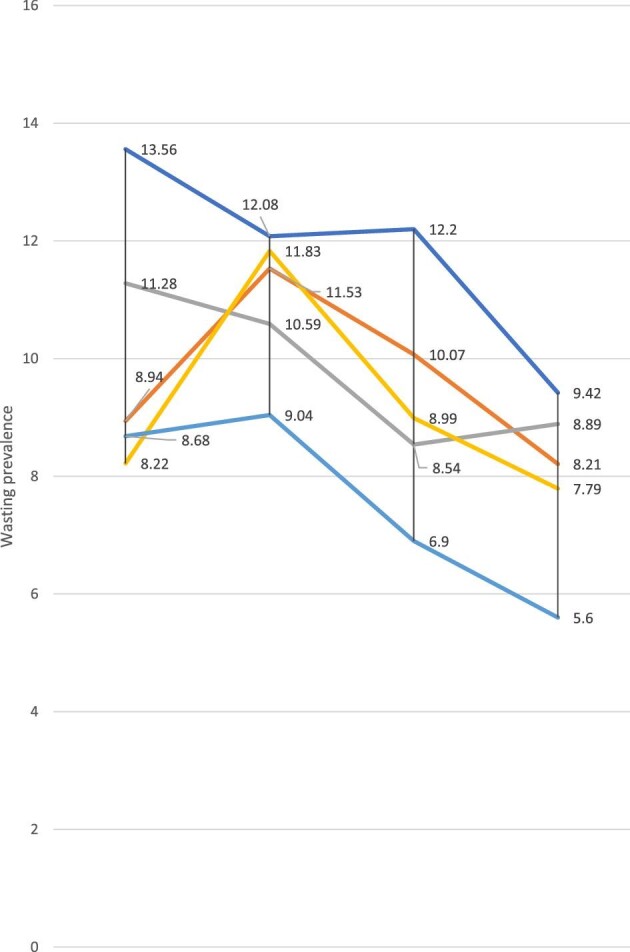
Prevalence of childhood wasting by wealth quintiles in Guinea: evidence from GDHS (1999–2012) and GMICS (2016).

Appendix 2 shows the magnitude and trends of childhood wasting across subpopulations from 1999 to 2016. We observed a lower prevalence among advantaged groups, such as richest, educated mothers, urban residents and from regions such as Mamou (in 2016). For example, significantly higher proportions of childhood wasting were found among children in quintile 1 (poorest) and quintile 2 (poorer) in 2012 and 2016 compared with quintiles 5 (richest) and quintile 4 (richer), respectively (Figure 1). The pattern of wasting was also found to be constant across populations in all wealth quintiles. For instance, the prevalence of childhood wasting among children in the poorest subpopulation group was higher by approximately 4.8 percentage points (pp) (95% CI 0.92 to 8.82) in 1999 compared with children in quintile 5 (richest wealth subgroup).

**Figure 2.**
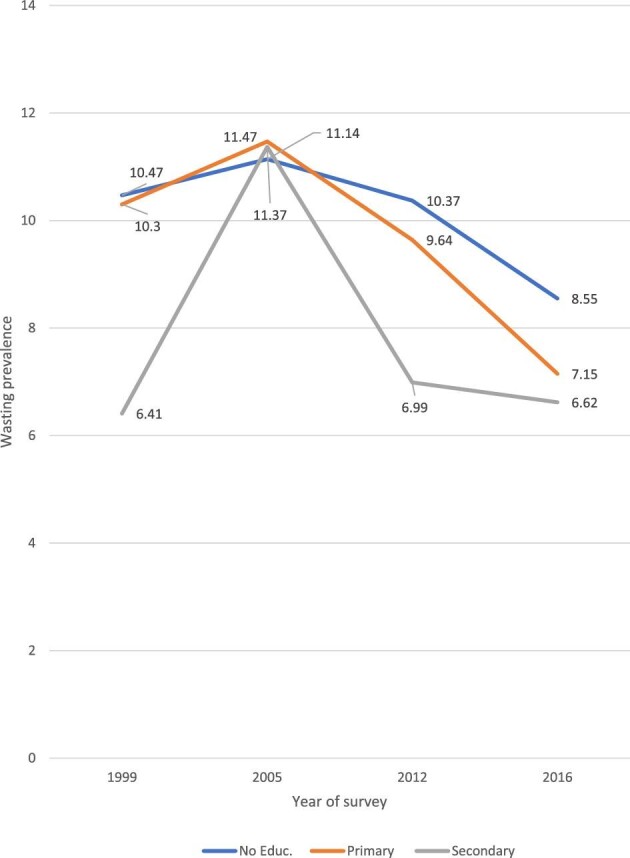
Prevalence of childhood wasting by maternal education status in Guinea: evidence from GDHS (1999–2012) and GMICS (2016).

We observed no big difference in the prevalence of childhood wasting across education subpopulations, particularly in 2005. Nonetheless, wasting was common among children whose parents have no education. Again, the pattern of wasting prevalence across education groups was constant (Figure 2).

**Figure 3.**
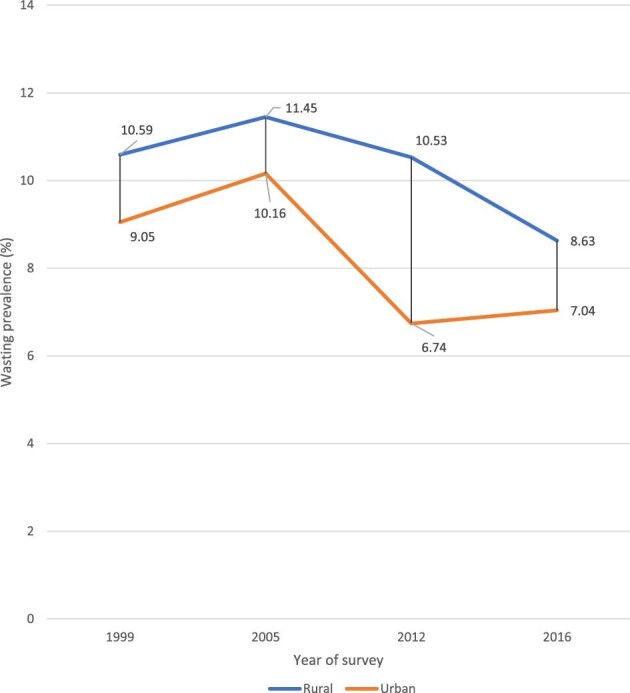
Prevalence of childhood wasting by place of residence in Guinea: evidence from GDHS (1999–2012) and GMICS (2016).

Although this study shows no profound difference in the prevalence of childhood wasting across the rural–urban subpopulation, a higher prevalence was observed among children from rural areas. For example, in 2016, the prevalence of wasting among rural children was 8.6 pp (95% CI 7.54 to 9.85), compared with 7.0 pp (95% CI 5.93 to 8.33) among urban children (Figure 3).

**Figure 4.**
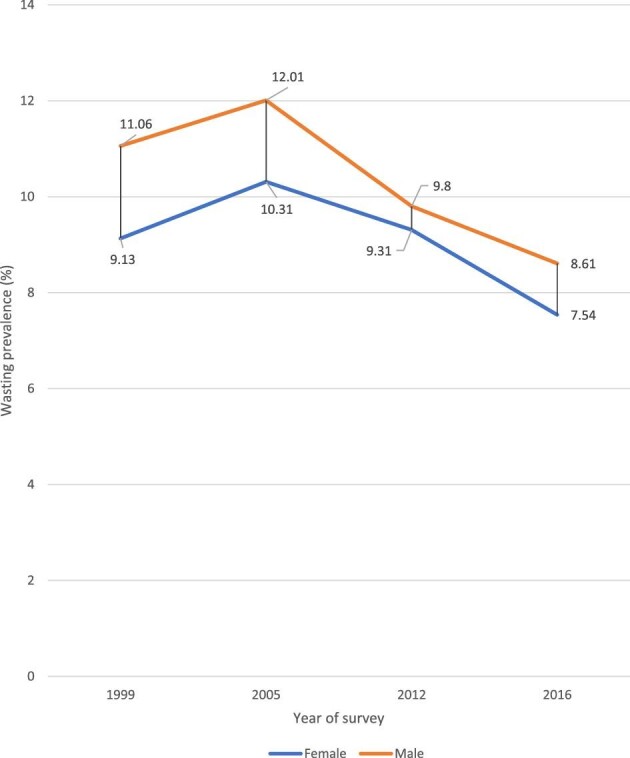
Prevalence of childhood wasting by child sex in Guinea: evidence from GDHS (1999–2012) and GMICS (2016).

Regarding a child's sex, a slight difference in the prevalence of childhood wasting was observed across all surveys. In 2016, for example, wasting prevalence among male children was 8.6 pp (95% UI 7.61–9.73) compared with 7.5 pp (95% CI 6.50 to 8.73) among female children (Figure 4).

**Figure 5.**
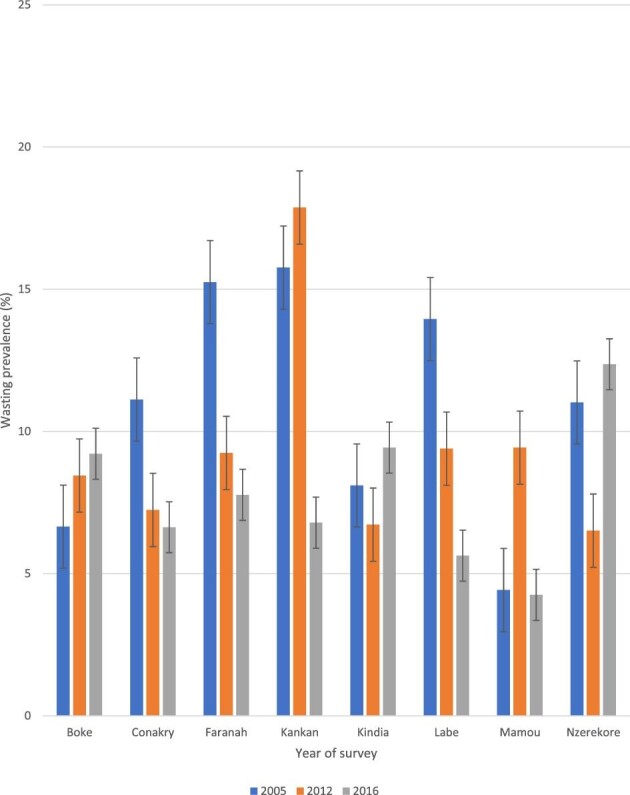
Prevalence of childhood wasting across subnational regions in Guinea: evidence from GDHS (2005–2012) and GMICS (2016).

Furthermore, we found significant differences in the prevalence of childhood wasting across subnational regions in all surveys. For example, in 2016 the prevalence of wasting among children living in the Nzérékoré region was higher by 8.1 pp (95% CI 4.85 to 11.36) when compared with children living in the Mamou region. The pattern of wasting prevalence varied over time across regions. In Kankan region, for instance, the prevalence of wasting decreased from 17.9 pp (95% CI 14.94 to 21.24) in 2012 to 6.8 pp (95% CI 5.27 to 8.72) in 2016. Meanwhile, in the Boke region, it remained fairly constant, as 8.5 pp (95% CI 6.03 to 11.72) was observed in 2012 and 9.2 pp (95% CI 7.53 to 11.22) was observed in 2016 (Figure 5).

For more detailed information on childhood wasting prevalence across different subpopulations, see Supplementary file 2.

### Magnitude and trends of socio-economic disparities

Table 1 shows socio-economic and geographic disparities in childhood wasting across subpopulations in Guinea from 1999 to 2016.

**Table 1.** Trends in socio-economic and area-based inequality in childhood wasting in Guinea: evidence from GDHS (1999–2016)

Dimension of inequality	Summary measure	1999, estimate (95% CI)	2005, estimate (95% CI)	2012, estimate (95% CI)	2016, estimate (95% CI)
Economic status	D	4.87 (0.92 to 8.82)	3.03 (−1.39 to 7.46)	5.30 (1.85 to 8.74)	3.81 (1.53 to 6.10)
	PAF	−14.24 (−34.94 to 6.46)	−19.07 (−44.46 to 6.30)	−27.86 (−49.86 to −5.86)	−30.73 (−46.22 to −15.25)
	PAR	−1.44 (−3.54 to 0.65)	−2.13 (−4.97 to 0.70)	−2.66 (−4.77 to −0.56)	−2.48 (−3.74 to −1.23)
	R	1.56 (0.98 to 2.13)	1.33 (0.75 to 1.91)	1.76 (1.06 to 2.47)	1.68 (1.11 to 2.24)
Education	D	4.05 (0.60 to 7.50)	−0.22 (−7.20 to 6.75)	3.37 (−0.20 to 6.95)	1.93 (−0.04 to 3.90)
	PAF	−36.71 (−68.19 to −5.23)	0 (−53.83 to 53.83)	−29.75 (−58.40 to −1.09)	−18.18 (−36.10 to −0.26)
	PAR	−3.71 (−6.90 to −0.53)	0 (−6.01 to 6.01)	−2.96 (−5.82 to −0.10)	−1.47 (−2.92 to −0.02)
	R	1.63 (0.79 to 2.47)	0.98 (0.37 to 1.58)	1.48 (0.75 to 2.20)	1.29 (0.92 to 1.65)
Residence	D	1.53 (−0.83 to 3.90)	1.29 (−1.64 to 4.23)	3.79 (1.60 to 5.98)	1.58 (−0.06 to 3.24)
	PAF	−10.58 (−26.64 to 5.47)	−9.07 (−29.15 to 11.00)	−29.5 (−45.04 to −13.95)	−12.95 (−23.55 to −2.34)
	PAR	−1.07 (−2.69 to 0.55)	−1.01 (−3.25 to 1.23)	−2.82 (−4.30 to −1.33)	−1.04 (−1.90 to −0.18)
	R	1.16 (0.87 to 1.46)	1.12 (0.81 to 1.43)	1.56 (1.12 to 1.99)	1.22 (0.96 to 1.48)
Sex	D	1.93 (−0.20 to 4.07)	1.70 (−1.28 to 4.68)	0.49 (−1.72 to 2.72)	1.06 (−0.45 to 2.59)
	PAF	−9.85 (−20.99 to 1.27)	−7.70 (−18.58 to 3.17)	−2.67 (−13.15 to 7.80)	−6.75 (−14.78 to 1.28)
	PAR	−0.99 (−2.12 to 0.12)	−0.86 (−2.07 to 0.35)	−0.25 (−1.25 to 0.74)	−0.54 (−1.19 to 0.10)
	R	1.21 (0.95 to 1.47)	1.16 (0.85 to 1.47)	1.05 (0.80 to 1.29)	1.14 (0.92 to 1.35)
Region	D	6.47 (2.82 to 10.12)	11.33 (5.03 to 17.64)	11.36 (7.03 to 15.69)	8.11 (4.85 to 11.36)
	PAF	−22.87 (−38.62 to −7.12)	−60.41 (−89.97 to −30.85)	−31.92 (−50.44 to −13.40)	−47.46 (−68.25 to −26.66)
	PAR	−2.31 (−3.91 to −0.72)	−6.75 (−10.05 to −3.44)	−3.05 (−4.82 to −1.28)	−3.84 (−5.52 to −2.15)
	R	1.82 (1.27 to 2.38)	3.56 (1.35 to 5.76)	2.74 (1.39 to 4.09)	2.90 (1.702 to 4.11)

### Economic inequality

Significant absolute and relative wealth-driven disparities in childhood wasting were observed in 2012 and 2016 using all four measures: D, PAF, PAR and R. The patterns of disparities from 2012 to 2016 were fairly constant. For instance, the PAR measure in 2012 (−2.66 [95% confidence interval {CI} −4.77 to −0.56]) and in 2016 (−2.48 [95% CI −3.74 to −1.23]) indicated significant absolute economic inequality in childhood wasting, with a fairly constant pattern over time. Similarly, the R measure in 2012 (1.76 [95% CI 1.06 to 2.47]) and 2016 (1.68 [95% CI 1.11 to 2.24]) indicated substantial relative wealth-driven inequality in childhood wasting over time.

### Education inequality

The result showed substantial absolute and relative education-related disparities by the complex measures (PAF and PAR), except in 2005. The simple measures (D and R) did not show education inequality across all surveys, except D in 1999. Generally the overall pattern of education inequality over the last 17 y was fairly constant, although the inequalities disappeared in 2005.

### Place of residence inequality

Complex measures (PAF and PAR) showed an urban–rural disparity in childhood wasting in 2012 and 2016. For instance, the PAF measure in 2012 and 2016 (−29.5 [95% CI −45.04 to −13.95] and −12.95 [95% CI −23.55 to −2.34], respectively) indicated relative disparities in childhood wasting. Moreover, the PAR measure in 2012 and 2016 (−2.82 [95% CI −4.30 to −1.33] and −1.04 [95% CI −1.90 to −0.18], respectively) indicated absolute residence disparities in childhood wasting.

### Sex-based inequality

The findings revealed no sex-related absolute or relative inequality in childhood wasting. For instance, the PAR measure in 1999, 2005, 2012 and 2016 (−0.99 [95% CI −2.12 to 0.12], −0.86 [95% CI −2.07 to 0.35], −0.25 [95% CI −1.25 to 0.74] and −0.54 [95% CI −1.19 to 0.10], respectively) supported the absence of sex-related absolute disparities across all the survey years.

### Subnational region inequality

Subnational region inequality in wasting was observed from 1999 to 2016 using both simple (D and R) and complex (PAF and PAR) measures, with a fairly constant pattern over time. For example, the PAR measures in 1999, 2005, 2012 and 2016 (−2.31 [95% CI −3.91 to −0.72], −6.75 [95% CI −10.05 to −3.44], −3.05 [95% CI −4.82 to −1.28], −3.84 [95% CI −5.52 to −2.15], respectively) indicated substantial absolute regional inequality in childhood wasting, with a fairly constant pattern over time. Similarly, the R measures in 1999, 2005, 2012 and 2016 (1.82 [95% CI 1.27 to 2.38], 3.56 [95% CI 1.35 to 5.76], 2.74 [95% CI 1.39 to 4.0] and 2.90 [95% CI 1.70 to 4.11], respectively) indicated significant relative regional disparities in childhood wasting, with a fairly constant pattern over time.

## Discussion

In this study, we assessed the prevalence of childhood wasting across different subpopulations as well as trends in socio-economic, sex and geographic inequalities in childhood wasting, using nationally representative GDHSs and GMICSs from 1999 to 2016. Our findings showed a childhood wasting prevalence of 10.1%, 11.2%, 9.6% and 8.1% in 1999, 2005, 2012 and 2016, respectively. Socio-economic and geographic inequalities in childhood wasting were found across all survey years, but varied on summary measures.^13,20^

Consistent with previous findings from studies in Nigeria.^13^ Ghana,^21^ Mozambique^22^ and SSA,^41^ though the magnitude was small, we found education-related inequality in childhood wasting favouring children born to educated mothers. Parental education, more specifically, maternal education, has been shown to have significant effects on child health.^22,42^ Health-seeking behaviour and utilization for promoting child health, including the nutrition status of children, are mostly practiced among educated mothers.^43,44^ Previous studies in Mozambique and Uganda showed that maternal education is significantly associated with childhood nutritional status.^22,42^ Maternal education may influence childhood nutrition in many ways, including decisions on the allocation of resources for household food consumption and other health-related issues.^45^

We further found pro-rich inequality in childhood wasting in Guinea over time. Previous studies in Ghana,^21^ Mozambique^22^ and SSA^41^ reported similar findings. Differences in household income level, areas of residence, parental education and occupational status and type could explain variations in wasting based on SES.^21,22,46^ Moreover, food security^47^ in the household, hygiene and sanitation practices and healthcare service accessibility and utilization^46^ are largely dependent on SES.^46,47^ This is supported by previous studies in Latin America and Nigeria,^48^ where improvement in the SES of women in areas such as education, conditional cash transfer, income level, food security and urbanization has significantly reduced childhood malnutrition, including wasting.^48^

Pro-urban inequalities in childhood wasting were observed, consistent with a previous study in Nigeria.^13^ Inequalities in the place of residence in childhood wasting have also been linked with differences in SES.^21,46^ Low SES, including poverty, illiteracy and unemployment, are more common in rural settings,^4^ and residents may be at risk of ill health.^41,49^ A higher prevalence of wasting among rural residents may therefore be seen as a deterioration in health conditions and poor access to healthcare services,^13^ consequently widening the urban–rural gap in health.^13^ Nonetheless, there is evidence that urban–rural disparities in child health could be reduced when socio-economic inequalities are addressed.^50^

Finally, we found significant regional inequality in childhood wasting across all the survey years, consistent with a previous study.^51^ The higher proportion of uneducated mothers^45^ and the urban–rural nature of the provinces^51,52^ in the country may explain the variation in childhood wasting prevalence across regions.^13,45,52^ There is evidence that wasting may be caused by a reduced intake of food,^53^ therefore the regional difference may be partly due to seasonal food insecurity that is related to natural disasters such as extreme poverty, crop damage by locusts and shifts in the practice of agriculture.^54,55^ A study conducted in Ethiopia confirmed a strong link between regional variations of malnutrition and dissimilarity in cultivated areas.^56^ The issue of food insecurity in Guinea is not surprising in light of the Ebola crisis. In 2013, 1 million of Guinea's 11.75 people were food insecure and 2.85 million were borderline food insecure. Food insecurity in Guinea varies between urban and rural residents, with rural residents being three times more likely to be food insecure. For example, in Faranah, 40.6% of households are facing food insecurity.^57^

However, food security does not always guarantee nutritional security, but it can be a precursor for nutritional improvements so long as the household properly manages the available food. Other factors such as infectious diseases might be another reason for regional variations in the magnitude of childhood wasting.^58,59^

### Strengths and limitations

This study has both strengths and limitations. First, the study assessed not only trends of inequality, but also trends of prevalence across different subpopulations using nationally representative data. Second, the study used the recommended WHO Health Equity Monitor database through HEAT software to analyse inequality. Third, we used simple and complex as well as absolute and relative summary measures to assess the inequality of different dimensions. By this approach, policymakers can view problems from different perspectives and provide appropriate interventions. The main limitation of this study was the inability to explore other factors related to disparities in wasting.

## Conclusions

Our study provides the first evidence of trends in socio-economic, sex and geographic-based inequalities in childhood wasting. With the exception of education-related inequality in 2005, we found absolute and relative socio-economic and geographic-related disparities in childhood wasting that favoured children from advantaged subpopulations, including rich/richest, educated mothers and children living in urban settings and regions such as Mamou in 2016. Although inequalities varied based on different summary measures, the pattern generally remained constant over time. Policies targeting disadvantaged populations need to be considered. This can help to ensure social protection, access to a healthy diet throughout the life course and universal and quality health services, which may in turn reduce wasting by addressing drivers such as poor hygiene and sanitation and household food insecurity that may be aggravated during emergencies, outbreaks of communicable diseases and disasters. Moreover, programs and interventions against childhood wasting need to be strengthened and prioritized for children of uneducated women, those from poor homes, rural residents and those in disadvantaged regions of Guinea. Nutrition education for disadvantaged mothers may have to highlight the need for inexpensive balanced and nutritious meals within their communities.

## Supplementary Material

ihac002_Supplemental_Files

## Data Availability

The datasets generated and/or analysed during the current study are available in the WHO's HEAT version 3.1 (https://www.who.int/gho/health_equity/assessment_toolkit/en/).
