# Supplementary material for: Time trends in socio-economic and geographic-based inequalities in childhood wasting in Guinea over 2 decades: a cross-sectional study
Source: Int Health. 2022 Feb 1;15(1):10–8. doi: 10.1093/inthealth/ihac002 (PMC9808518; doi:10.1093/inthealth/ihac002)
Supplement: ihac002_Supplemental_Files [file ihac002_supplemental_files.zip › Suppl 1.docx]

**Supplementary file 1.** Summary measures and the methods for calculating the summary measures

**Calculation of summary measures**

Calculation of summary measures varied based on dimension inequality. That means it varied for ordered, non-ordered and binary dimension of inequality. D is calculated as the difference between two subgroups: for instance, for education, D was calculated as wasting prevalence in “uneducated” group minus wasting prevalence in “secondary education” group. Economic status was calculated as wasting prevalence in the poorest group minus wasting prevalence in the richest group. Relatedly, D was calculated as wasting prevalence in rural minus wasting in urban populations with respect to place of residence, male minus female for sex and region with the highest estimate minus the one with the lowest estimate in relation to subnational region. Except divide for ratio and minus for difference, the calculation and references are same.

The change in childhood wasting estimate for the reference subgroup, yref, and the national average of wasting prevalence was used to compute the PAR. For ordered dimensions the most advantaged sub-group describes y_ref_ , which in our case are the secondary school and above subgroups for education and richest sub-group for economic status and for binary dimensions such as sex and place of residence, y_ref_ refers to the subgroup which has the lowest estimate, which in our case was female and urban subgroups respectively. For non-ordered dimensions like subnational region, y_ref_ points out the subgroup or region with the lowest estimate. PAF were computed by dividing the PAR by the national average μ and multiplying the fraction by 100 (PAF = [PAR / μ] * 100). Greater values of absolute PAR and PAF values denoted higher levels of inequality whereas zero indicates absence of inequality.

**Interpretation of summary measures**

If there is no inequality, D takes the value zero. Greater absolute values indicate higher levels of inequality. Positive values indicate a higher concentration of wasting among the disadvantaged and negative values indicate a higher concentration among the advantaged.

If there is no inequality, R takes the value one. It takes only positive values (larger or smaller than 1). The further the value of R from 1, the higher the level of inequality.

PAR and PAF takes negative values for adverse health outcome indicators such as wasting. The larger the absolute value of PAR, the higher the level of inequality. PAR is zero if no further improvement can be achieved, i.e. if all subgroups have reached the same level of wasting prevalence as the reference subgroup.
