# Supplementary material for: Time trends in socio-economic and geographic-based inequalities in childhood wasting in Guinea over 2 decades: a cross-sectional study
Source: Int Health. 2022 Feb 1;15(1):10–8. doi: 10.1093/inthealth/ihac002 (PMC9808518; doi:10.1093/inthealth/ihac002)
Supplement: ihac002_Supplemental_Files [file ihac002_supplemental_files.zip › Suppl 2.docx]

**Supplementary file 2.** Trends of childhood wasting across socio-economic and area-based subgroups in Guinea: Evidence from Guinea demographic and health surveys (1999-2016)

| **Dimension of Inequality** | **Subgroup** | | **1999** | | **2005** | | **2012** | | **2016** | |
| --- | --- | --- | --- | --- | --- | --- | --- | --- | --- | --- |
|  |  |  | **Estimate (95% UI)** | **Pop^n^** | **Estimate (95% UI)** | **Pop^n^** | **Estimate (95% UI)** | **Pop^n^** | **Estimate (95% UI)** | **Pop^n^** |
| Economic status | Quintile 1 (poorest) | | 13.56 (10.79, 16.90) | 599 | 12.08 (9.36, 15.45) | 669 | 12.20 (9.89, 14.96) | 793 | 9.42 (7.93, 11.15) | 1463 |
|  | Quintile 2 | | 8.94 (6.97, 11.40) | 529 | 11.53 (9.11, 14.49) | 546 | 10.07 (7.61, 13.20) | 800 | 8.21 (6.54, 10.25) | 1587 |
|  | Quintile 3 | | 11.28 (8.78, 14.37) | 556 | 10.59 (7.88, 14.08) | 585 | 8.54 (6.24, 11.60) | 710 | 8.89 (7.21, 10.92) | 1444 |
|  | Quintile 4 | | 8.22 (6.43, 10.47) | 644 | 11.83 (8.83, 15.66) | 483 | 8.99 (6.93, 11.58) | 714 | 7.79 (6.32, 9.57) | 1396 |
|  | Quintile 5 (richest) | | 8.68 (6.44, 11.62) | 582 | 9.04 (6.25, 12.91) | 359 | 6.90 (4.88, 9.66) | 512 | 5.60 (4.17, 7.48) | 1161 |
| Education | No Educ. | | 10.47 (9.27, 11.79) | 2403 | 11.14 (9.57, 12.93) | 2332 | 10.37 (8.99, 11.93) | 2455 | 8.55 (7.66, 9.53) | 5117 |
|  | Primary | | 10.30 (7.24, 14.44) | 278 | 11.47 (6.29, 20.03) | 198 | 9.64 (6.53, 14.01) | 350 | 7.15 (5.39, 9.43) | 924 |
|  | Secondary | | 6.41 (3.83, 10.52) | 231 | 11.37 (6.11, 20.18) | 111 | 6.99 (4.34, 11.08) | 300 | 6.62 (5.07, 8.59) | 1010 |
| Place of residence | Rural | | 10.59 (9.26, 12.08) | 2035 | 11.45 (9.65, 13.54) | 2075 | 10.53 (9.13, 12.12) | 2627 | 8.63 (7.54, 9.85) | 4650 |
|  | Urban | | 9.05 (7.29, 11.19) | 878 | 10.16 (8.13, 12.64) | 567 | 6.74 (5.30, 8.54) | 903 | 7.04 (5.93, 8.33) | 2402 |
| Sex | Female | | 9.13 (7.73, 10.75) | 1408 | 10.31 (8.42, 12.57) | 1304 | 9.31 (7.83, 11.03) | 1709 | 7.54 (6.50, 8.73) | 3444 |
|  | Male | | 11.06 (9.61, 12.71) | 1505 | 12.01 (9.99, 14.38) | 1338 | 9.80 (8.34, 11.48) | 1821 | 8.61 (7.61, 9.73) | 3608 |
| Subnational region | Lower Guinea | Boké | 8.95 (6.81, 11.68) | 659 | 6.65 (4.85, 9.06) | 285 | 8.45 (6.03, 11.72) | 361 | 9.21 (7.53, 11.22) | 877 |
|  | Central  Guinea | Conakry | 14.28(11.26, 17.95) | 494 | 11.12 (7.33, 16.50) | 210 | 7.24 (4.98, 10.40) | 448 | 6.63 (5.12, 8.55) | 1122 |
|  | Upper Guinea | Faranah | 11.41 (8.86, 14.56) | 461 | 15.25 (12.10, 19.05) | 242 | 9.24 (6.08, 13.81) | 335 | 7.77 (5.82, 10.29) | 583 |
|  | Forest Guinea | Kankan | 7.81 (6.38, 9.52) | 847 | 15.76 (10.66, 22.67) | 458 | 17.87 (14.94, 21.24) | 626 | 6.79 (5.27, 8.72) | 1285 |
|  | Conakry | Kindia | 10.33 (7.69, 13.75) | 449 | 8.10 (5.15, 12.51) | 393 | 6.72 (4.58, 9.76) | 546 | 9.43 (6.30, 13.88) | 919 |
|  | NA | Labe | NA | NA | 13.95 (10.14, 18.90) | 241 | 9.39 (6.48, 13.41) | 309 | 5.63 (4.19, 7.52) | 580 |
|  | NA | Mamou | NA | NA | 4.42 (2.68, 7.21) | 169 | 9.43 (6.57, 13.35) | 240 | 4.25 (3.01, 5.96) | 555 |
|  | NA | Nzerekore | NA | NA | 11.02 (8.23, 14.61) | 642 | 6.51 (4.08, 10.22) | 662 | 12.36 (9.72, 15.58) | 1128 |

Notes: Pop^n^: population, NA: Not applicable, UI: Uncertainty Interval
